# Prognostic Evaluation Based on Dual-Time ^18^F-FDG PET/CT Radiomics Features in Patients with Locally Advanced Pancreatic Cancer Treated by Stereotactic Body Radiation Therapy

**DOI:** 10.1155/2022/6528865

**Published:** 2022-07-14

**Authors:** Fei Wang, Chao Cheng, Shengnan Ren, Zhongyi Wu, Tao Wang, Xiaodong Yang, Changjing Zuo, Zhuangzhi Yan, Zhaobang Liu

**Affiliations:** ^1^Institute of Biomedical Engineering, School of Communication and Information Engineering, Shanghai University, Shanghai 200444, China; ^2^Department of Medical Imaging, Suzhou Institute of Biomedical Engineering and Technology, Chinese Academy of Sciences, Suzhou 215163, China; ^3^Department of Nuclear Medicine, Changhai Hospital, Naval Medical University, Shanghai 200433, China

## Abstract

**Background:**

^18^F-FDG PET/CT is widely used in the prognosis evaluation of tumor patients. The radiomics features can provide additional information for clinical prognostic assessment.

**Purpose:**

Purpose is to explore the prognostic value of radiomics features from dual-time ^18^F-FDG PET/CT images for locally advanced pancreatic cancer (LAPC) patients treated with stereotactic body radiation therapy (SBRT).

**Materials and Methods:**

This retrospective study included 70 LAPC patients who received early and delayed ^18^F-FDG PET/CT scans before SBRT treatment. A total of 1188 quantitative imaging features were extracted from dual-time PET/CT images. To avoid overfitting, the univariate analysis and elastic net were used to obtain a sparse set of image features that were applied to develop a radiomics score (Rad-score). Then, the Harrell consistency index (C-index) was used to evaluate the prognosis model.

**Results:**

The Rad-score from dual-time images contains six features, including intensity histogram, morphological, and texture features. In the validation cohort, the univariate analysis showed that the Rad-score was the independent prognostic factor (*p* < 0.001, hazard ratio [HR]: 3.2). And in the multivariate analysis, the Rad-score was the only prognostic factor (*p* < 0.01, HR: 4.1) that was significantly associated with the overall survival (OS) of patients. In addition, according to cross-validation, the C-index of the prognosis model based on the Rad-score from dual-time images is better than the early and delayed images (0.720 vs. 0.683 vs. 0.583).

**Conclusion:**

The Rad-score based on dual-time ^18^F-FDG PET/CT images is a promising noninvasive method with better prognostic value.

## 1. Introduction

Pancreatic cancer is a malignant tumor. Although the prognosis of patients with pancreatic cancer patients has improved due to early diagnosis and treatment methods, the 1-year and 5-year survival rate of patients is about 15% and 9%, respectively [1, 2]. Surgical resection can effectively prolong the survival of patients with pancreatic cancer. However surgical resection can only be performed in about 20% of patients, and most patients with locally advanced pancreatic cancer (LAPC) cannot be treated with surgery, and their median survival time was only 6–14 months [3]. For these LAPC patients who cannot tolerate surgical treatment, stereotactic body radiation therapy (SBRT) was a commonly used treatment [4, 5]. However, not all patients with LAPC can benefit from SBRT [6], so it is very important to predict the outcome of treatment based on the patient's information.

Compared with most normal organs and tissues, the uptake of ^18^F-FDG in tumor areas increased significantly. Therefore, ^18^F-FDG PET/CT images have been widely used in tumor imaging [7]. In terms of pancreatic cancer, ^18^F-FDG-PET-CT scanning plays an important role in diagnosis, staging, and efficacy evaluation [8]. Some quantitative parameters based on PET imaging were shown to have some prognostic value in cancer patients, such as maximum and mean standard intake values (SUVmax and SUVmean). However, for pancreatic cancer [9–12], most studies show that the standardized uptake value (SUV) cannot be used as a prognostic indicator of survival. However, for metabolic tumor volume (MTV) and total disease glycolysis (TLG), findings demonstrated that they were significantly associated with the OS of patient. It must be emphasized that the importance of MTV and TLG in predicting the prognosis of pancreatic cancer varies in different clinical trials, which may be due to different criteria for tumor zoning. In addition, some researchers use the retention index (RI) from the dual-time ^18^F-FDG PET/CT images for prognostic assessment. Gupta et al. [13] found that RI > 18.7% was positively associated with poorer survival in patients with pancreatic lesions. Saga et al. [14] showed that pancreatic cancer patients with a high RI (RI > 10%) had a longer survival time. The effectiveness of RI needs further experimental verification. Therefore, there was a need for new and robust imaging features to predict the prognosis of pancreatic cancer patients.

Tumor heterogeneity is an important factor affecting the prognosis of patients after treatment [15]. Radiomics, which involves the extraction of quantitative features from medical images to provide a comprehensive characterization of the entire tumor, reflects the spatial relationship and heterogeneity of voxel intensities within tumors [16–18]. Currently, studies have shown that radiomics features can be used for cancer diagnosis, prognosis, and preoperative staging [19–21]. Some studies have shown that texture features from PET/CT images can be used as predictors for prognosis. For example, Yue et al. [22] studied the relationship between texture features from PET images before and after chemotherapy and the OS of patients with pancreatic cancer. The results showed that some texture features were prognostic factors of patients. Cui et al. [23] showed that texture features from early PET images showed better prognostic value than conventional PET features in patients treated with SBRT. And studies have shown that continuously dynamic ^18^F-FDG PET/CT parameters can be used as predictors of patient survival [13, 24]. However, it was difficult to achieve 60-minute, dynamic ^18^F-FDG PET/CT scans for patient prognosis. Recently, the dual-time FDG-PET/CT (early and delayed images) has been shown to be helpful in distinguishing benign from malignant pancreatic lesions [19, 25]. In general, the uptake of ^18^F-FDG in malignant tissues continues to increase over time [26, 27]. Therefore, the researchers believe that dual-temporal PET/CT images contain more prognostic information for patients. There, we assumed that the radiomics score (Rad-score) from dual-time static ^18^F-FDG PET/CT images can replace dynamic imaging to a certain extent, and predict the prognosis of patients according to the changes in tumor texture.

In this study, we hypothesized that the radiomics score (Rad-score) calculated by a linear combination of the radiomics features from dual-time images could be the reference indicator for the prognosis of LAPC patients. We evaluated whether the Rad-score from dual-time imaging had a better prognostic value than the Rad-score from early or delayed images. The ultimate aim of this retrospective study was to explore the role of the radiomics features from dual-time ^18^F-FDG PET/CT images in predicting the prognosis of LAPC patients treated with SBRT.

## 2. Patients and Methods

### 2.1. Patients

This retrospective study was approved by the Ethics Committee of Changhai Hospital, and informed consent was given to all participants. The criteria for patient inclusion were (a) confirmation of pancreatic cancer on pathological examination of the patient after PET/CT scan; (b) available dual-time ^18^F-FDG PET/CT images; and (c) underwent SBRT treatment. The exclusion criteria were (a) other malignant tumors; (b) death due to diseases other than pancreatic cancer during the follow-up period; and (c) a metal positioning mark implanted in the tumor lesion. Finally, a total of 70 patients who underwent dual-time ^18^F-FDG PET/CT examinations in our hospital between January 2012 and January 2018 were identified and included in the study. The survival time of patients was determined from the date of the FDG-PET examination to the last follow-up examination in Changhai Hospital or the patient's death. Some of the data in this study have been reported [9].

### 2.2. PET/CT Imaging Protocols

The dual-time ^18^F-FDG PET/CT images data of the patients were collected on a Biograph tripoint 64-layer 52-ring HD PET/CT scanner (Siemens, Germany). Before the whole-body scan, the patients were required to fast for at least 6 hours. When their blood sugar was lower than 11.1 mmol/L, ^18^F-FDG at the dose of 3.70∼5.55 MBq/kg was injected, and the early scanning was started 50–60 minutes after the injection. The whole-body PET scan covers 5–6 beds, with an acquisition time per bed of about 2.5 minutes, a spatial resolution of 4.07 × 4.07 mm^2^, and a scan thickness of 3 mm. The parameters of the CT scan were a current of 170 mA, a voltage of 120 kV, a spatial resolution of 0.98 × 0.98 mm^2^, and a scan thickness of 3 mm. The PET and CT image matrix size were 168 × 168 and 512 × 512, respectively. After 120–150 minutes, delayed scanning was started. The delayed PET/CT images only contain the head to tail of the pancreas. Patients were required to breathe shallowly during PET/CT scans to reduce the impact of breathing exercises.

### 2.3. Image Analysis

**Figure 1:**
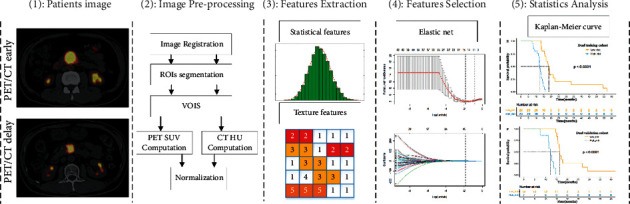
The workflow of radiomics analysis for prognosis. (a) Dual-time ^18^F-FDG PET/CT image data construction, (b) data preprocessing, including image registration, lesion segmentation, and data normalization, (c) features extraction, including statistical, morphological, and texture features, (d) features selection, including the univariate analysis and elastic net, (e) statistics analysis.

The radiomics workflow is shown in Figure 1. For image preprocessing, we used the 3D Slicer (version 4.10.2) to resample the original PET image and co-register it with the corresponding CT images [28, 29]. Under the guidance of the PET images, two radiologists with more than 10 years of clinical experience outlined the tumor contour on the CT images. Then the voxels of the CT were clipped to [−10, 100] Hounsfield Units to reduce the interference of fat and other factors on texture features [30, 31]. For the PET images, a classical normalization factor (body weight) was used to convert the voxel values to SUV values [32], and a square root transformation was used to reduce noise. Finally, the voxel values of PET and CT images were normalized to [0, 255].

The feature extraction algorithm was applied in MATLAB (The MathWorks, Inc. Natick, MA, USA). Before extracting the three-dimensional radiomics features, firstly, the volumes of interests (VOIs) were obtained by performing the nearest cubic trilinear interpolation on all ROI areas in the *Z*-axis direction. Then the spatial resolution of CT and PET images was resampled to 1 × 1 × 1 mm^3^ by cubic trilinear interpolation. Finally, a total of 1188 features were extracted from the early and the delayed PET/CT images, respectively, including three groups: texture features, shape features, and wavelet features (Supplementary Table 1).

### 2.4. Feature Selection

Before features analysis, each radiomics feature value was independently normalized by subtracting its mean and then dividing by its standard deviation. To improve the reproducibility and robustness of the optimal features, first, we used the intra-class correlation coefficient (ICC) to weed out features with a correlation lower than 0.75. Second, the univariate Cox regression analysis selection was used to select features that were significantly associated with patients' overall survival time (*p* < 0.05). Finally, we used elastic nets [33] for final feature screening and repeat this process 100 times, recording the features selected each time. To calculate the Rad-score, we then counted the frequency of the features and included the top six features in the final feature set. Then we retrained the multivariate Cox regression model [34] with the final feature subset in the training cohort and get the corresponding weights. The Rad-score is defined as the sum of the weights of each feature, which will be used in subsequent related experiments. The specific calculation formula is shown as follows:

(1) $$\begin{array}{c} \text{Rad}-\text{score}=\sum {\text{Features}}_{i}*{\text{Weights}}_{i}, \end{array}$$

where Features_*i*_ represent the value of selected features and Weights_*i*_ represent the corresponding weight of these features in the Cox regression model.

### 2.5. Statistics Analysis

To evaluate the effectiveness of the proposed Rad-score, first, patients were divided into a high-risk group and a low-risk group using the median of the Rad-score in the training cohort. And the Kaplan–Meier survival analysis with a log-rank test was used to analyze significant differences in low-risk and high-risk groups. Second, the Rad-score was used to build the prognosis model based on Cox proportional hazard regression analysis. The average *C*-index for three-fold cross-validation was used to evaluate the performance of the prognosis model. The Wilcoxon rank-sum test was used to evaluate whether there are significant differences between different Rad-score. Finally, the univariate and multivariate Cox regression analyses were used to analyze whether the candidate parameters are independent risk prediction factors in all data. All the above code was implemented in R (version 3.6.3) software.

## 3. Results

### 3.1. Baseline Clinical Information of the Patients

A total of 70 LAPC patients received early and delayed ^18^F-FDG PET/CT scans before SBRT treatment, which were randomly divided into a training cohort and a validation cohort at a ratio of 2:1, with 46 patients in the training cohort and 24 patients in the validation cohort. The baseline clinical information is shown in Table 1. Female patients accounted for about 38.6, with an average age of 65.7 ± 8.59 years. All patients received SBRT and underwent ^18^F-FDG PET/CT scan before treatment. There was no significant difference among the clinical variables between the training and validation cohorts.

### 3.2. Features Selection

To select more robust features, 216 features with ICC less than or equal to 0.75 were eliminated through inter-observer analysis. Then, we incorporated the remaining 972 features into the subsequent resampling experiment. Finally, we selected the six most frequent features to retrain the final Cox regression. The categories of these six features and their weights in the retrained Cox model are given in Table 2. The Rad-score was then built that was weighted by their respective coefficients in the Cox regression model and used as a risk predictor for OS.

### 3.3. Early PET/CT Images Analysis

**Figure 2:**
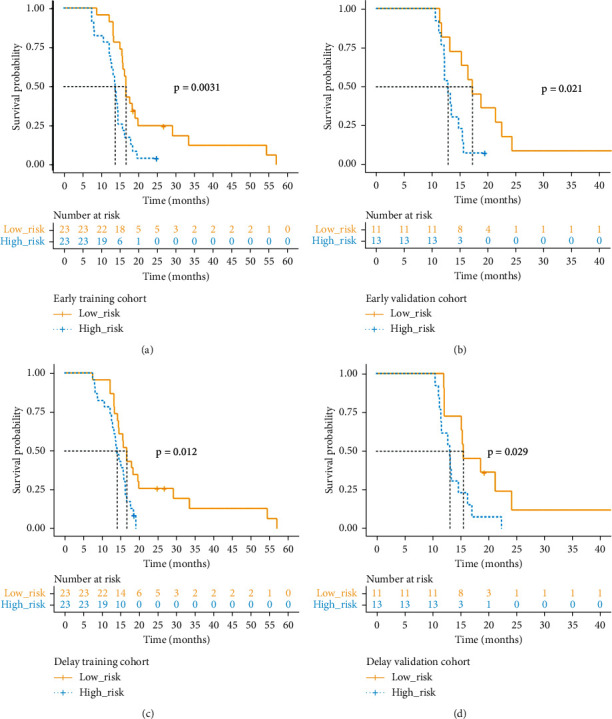
The Kaplan–Meier curve represents the OS of LAPC stratified according to the median value of the Rad-score in the training cohort (early images (a) and (b), delayed images (c) and (d)). The log-rank *p*-value is shown on the right side of each graph.

For early image analysis, according to the median Rad-score in the training cohort, patients were divided into high-risk groups and low-risk groups. There was a significant difference in the OS between the high-risk group and the low-risk group (*p* < 0.05) in the training and the validation cohorts. And the Kaplan–Meier curve for the Rad-score is shown in Figures 2(a) and 2(b). In terms of the prognostic accuracy of the prognostic model, the Rad-score calculated from the early image reached 0.683 ± 0.016 in the validation cohort (0.726 ± 0.030, training cohort).

### 3.4. Delayed PET/CT Images Analysis

For delayed image analysis, the Kaplan–Meier curves of the high-risk group and the low-risk group are shown in Figures 2(c) and 2(d). The OS of patients in the low-risk group was significantly better than that of the high-risk group (*p* < 0.05 in training and validation cohorts). We constructed a prognosis model based on the Rad-score in the training cohort, and its survival prediction accuracy in the validation cohort reached 0.583 ± 0.007 (0.653 ± 0.006, training cohort).

### 3.5. Dual time PET/CT Images Analysis

**Figure 3:**
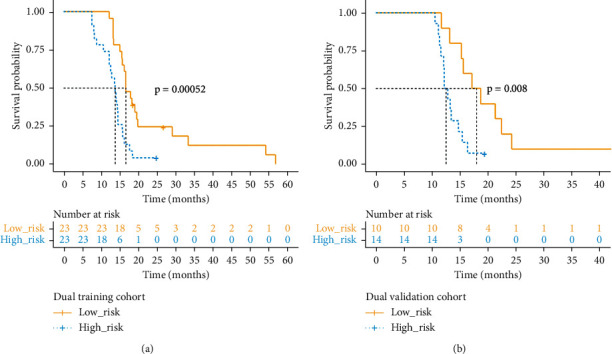
Kaplan–Meier survival curves and risk group stratification based on dual-time images.

The Kaplan–Meier curve of the Rad-score for high-risk and low-risk groups was seen in both training and validation cohorts (Figures 3(a) and 3(b), *p* < 0.05). In the training cohort, there was a significant difference in the survival time of patients between the low-risk group and those in the high-risk group, which was verified in the validation cohort (*p* < 0.01). The *C*-index of the dual-time model based on the Rad-score was 0.720 ± 0.031 in the validation cohort (0.729 ± 0.031, training cohort). In addition, the results of the Wilcoxon rank-sum test show that there are significant differences between models based on dual-time images and single-time image models (*p* < 0.05, Supplementary Table 2). Furthermore, we compared the prediction accuracy of several major radiomic features used in the Rad-score. The results show that the Rad-score has better prediction performance.

To determine the independent risk factors for patients treated with SBRT, we performed the univariate and multivariate Cox analysis of the Rad-score from dual-time images, clinical information, and conventional PET features (SUV, MTV, and TLG) in all data. The results are shown in Table 3. According to the univariate Cox regression analysis, the Rad-score from dual-time images was significantly correlated with the OS (*p* < 0.001) in the validation cohort. And higher SUVmax, SUVmean, MTV, and TLG from the delayed images were significantly correlated with shorter OS (*p*=0.07,  0.048,  0.003,  0.001), and clinical factors such as T-stage, N-stage, dose, and chemotherapy were also significantly correlated with OS (*p* < 0.05). On the other hand, with a multivariate Cox regression analysis, the Rad-score from dual-time images was the only independent prognostic factor (*p* < 0.001).

## 4. Discussion

The purpose of this study was to explore the predictive value of radiomics features from dual-time images on the prognosis of LAPC patients treated with SBRT. We found that the Rad-score from dual-time ^18^F-FDG PET/CT images can be used to predict the prognosis of LAPC patients, and can achieve the prognostic stratification with OS of patients. This result has clinical significance for promoting the precise treatment of LAPC.

The radiomics has attracted a lot of attention in its ability to noninvasively analyze tumor heterogeneity; and provides a viable tool for patient prognosis. In this study, we conducted a two-stage experimental setup to explore the best model for a prognosis for patients with pancreatic cancer. First, we use 594 radiomics features extracted from early or delayed imaging to develop a single-time prognostic model, and then we combined the radiomics features extracted from dual-time PET/CT images to develop a dual-time prognostic model. In these two stages, the six radiomics features with the highest frequency were used to calculate the Rad-score. Univariate analysis showed that the Rad-score of both early and delayed images was significantly correlated with the OS of LAPC patients (*p* < 0.01, HR: 3.00 and 2.70). This result indicated that both early and delayed PET/CT images contain patient prognostic information [13, 23]. The Rad-score from dual-time images is also significantly correlated with the OS of patients (*p* < 0.01, HR: 2.35). The *C*-index of the model based on dual-time images was significantly higher than that of the model based on early and delayed images (*p* < 0.05, Wilcoxon rank-sum test). At the same time, it must be pointed out that there are differences in the division of high-risk and low-risk groups according to the Rad-score of single-time images, while an accurate grouping can be obtained by analyzing the Rad-score of dual-time images. These results showed that dual-time PET/CT images can provide more prognostic information and offset the limitations of single-time PET/CT images [13]. This may be related to the kinetics of tumor uptake. As the uptake time increases, the uptake of ^18^F-FDG by malignant tumor tissues will increase significantly [26, 27].

In this study, the Rad-score from dual-time images includes three types of features such as shape feature, first-order gray statistics feature, and texture feature. Different types of texture features can reflect the inherent heterogeneity of tumors from different angles [35, 36]. In addition, the optimal features include both original texture features and texture features based on wavelet transform (wavelet coefficients: HHH = highpass filter + highpass filter + highpass filter), which reflect the heterogeneity of tumors on different spatial scales. On the one hand, it must be pointed out that when these six radiomics features were used alone, none of the features can predict patient survival better than the Rad-score, indicating the complementarity of information between different features. On the other hand, these characteristics are also significantly related to the OS of patients. The solidity reflects the complexity of the homogenous region of the tumor. The smaller the value, the higher the complexity of the tumor and the worse the prognosis of the patients [37]. The gray level difference statistics (GLDS) calculates the contrast of the image and reflects the roughness of the texture. The contrast [38] (PET, GLDS, and HHH) shows that the metabolic changes in the lesions in PET images have a strong resolution, the functional metabolic changes in the lesions are larger, and the texture is coarser in patients with poor prognosis. The Busyness (Neighborhood Gray-Tone Difference Matrix (NGTDM) and PET) measures the change from pixel to the adjacent pixel. A high value of business indicates that the intensity between a pixel and its neighborhood changes rapidly, indicating that the more complex the tumor is in patients with poor prognosis. The correlation and the energy from the gray-level size zone matrix (GLSZM) in CT images quantify the degree of nonuniformity of the gray level in images. The patients with a better prognosis showed better texture consistency.

The clinicopathological parameters, including T-stage, chemotherapy, and dose were found to be strong predictors of prognosis in LAPC patients receiving SBRT in the univariate Cox analysis. However, the above indicators did not show prognostic value for patients in the multivariate Cox analysis study. For the conventional PET features, the TLG from delayed PET/CT images is significantly associated with a poor prognosis. This reaffirms the fact that the metabolic tumor volume combined with the tumor range is a better predictor of patient survival. However, in this study, the TLG of the early images does not correlate with the OS. One possible reason is that compared with the early images, delayed images may better reflect the uptake of ^18^F-FDG by malignant tumors. This result is different from our previous research [9]. One possible reason is that the tumor contour is outlined in different ways, and another possible reason is the difference in the number of patients.

There are some limitations to this study. First, the samples in this study were from a single center, the sample size available for analysis was small, and the potential of selection bias cannot be ruled out, which limits the accuracy and reliability of the results. Therefore, we hope that the results of this study can be repeated using larger datasets and multiple centers in the future. Second, the ROI/VOI was drawn manually, which is very time-consuming and inconvenient, and the predicted performance may be sensitive to the ROI/VOI depicting pancreatic lesions. In future research, automatic segmentation or semi-automatic segmentation could be achieved through the application of deep learning.

## 5. Conclusion

In conclusion, the Rad-score obtained from dual-time ^18^F-FDG PET/CT images reflects the heterogeneity of intertumoral metabolism from different aspects. It is a powerful predictor of survival for patients with locally advanced pancreatic cancer treated with SBRT. The radiomics analysis of dual-time PET/CT images can help patients choose the appropriate treatment plan and realize precision medicine.

## Figures and Tables

**Table 1:** Clinical characteristics of the patients in the training cohort and the validation cohort. #Data are the number of patients and data in parentheses are the ratio.

Variable	Training cohort (*n* = 46)	Validation cohort (*n* = 24)	*p* value
Age (years)^*∗*^	68 (26, 82)	67 (45, 84)	0.985

Sex^#^			0.156
Male	31 (67.4%)	12 (50.0%)	
Female	15 (32.6%)	12 (50.0%)	

*T* stage^#^			0.345
1	2 (4.3%)	1 (4.2%)	
2	12 (26.1%)	5 (20.8%)	
3	15 (32.6%)	13 (54.2%)	
4	17 (32.7%)	5 (20.8%)	

*N* stage^#^			0.102
0	30 (65.2%)	10 (41.7%)	
1	16 (34.8%)	14 (58.3%)	

ECOG^#^			0.458
0	8 (17.4%)	7 (29.2%)	
1	23 (50.0%)	9 (37.5%)	
2	15 (32.6%)	8 (33.3%)	

CA19-9^*∗*^	365 (2, 2125)	132 (2, 1200)	0.504

Longest diameter (cm)^*∗*^	3.6 (1.5, 7.5)	3.9 (1.0, 6.3)	0.268

Location^#^			0.783
Head	34 (73.9%)	17 (70.8%)	
Body/distal	12 (26.1%)	7 (29.2%)	

Chemotherapy^#^			0.312
0	29 (63.0%)	18 (75.0%)	
1	17 (37.0%)	16 (25.0%)	

Dose^*∗*^	37.2 (30, 46.8)	36 (30, 46.8)	0.454

OS (month)^*∗*^	15.4 (7.5, 56.8)	14 (10.6, 43.9)	0.449

^*∗*^Data are the median and data in parentheses are the range. Chi-square test and Mann–Whiney *U* test are used to compare the difference between categorical and continuous variables in the training cohort and the validation cohort, respectively. ECOG, eastern cooperative oncology group; CA19-9, carbohydrate antigen 19–9; dose, radiotherapy dose (Gy).

**Table 2:** Radiomics features were selected via the elastic net and the corresponding weights in retraining the Cox regression model.

Feature name	Categories	Modality	Time	Weights		
				Early	Delay	Dual
Solidity	Shape	—	Early	−3.7884	—	−2.0451
Contrast	GLDS (HHH)	CT		0.3654		1.4669
Contrast	GLDS (HHH)	PET		0.5546		1.9619
Energy	GLDS (LLL)	CT		6.4719		—
Contrast	GLDS (HHH)	PET		−0.1456		—
Energy	GLCM (LLH-LHL-HLL)	CT		−0.1552		—
Busyness	NGTDM (LLL)	PET	Delay	—	3.5622	2.3483
Entropy	Histogram (original)	CT			0.8987	2.5573
Gray level nonuniformity	GLSZM (LLH-LHL-HLL)	CT			3.1435	3.1575
Busyness	NGTDM (original)	PET			−1.2738	—
Entropy	GLDS (original)	CT			0.3695	—
Mean	GLDS (LLL)	PET			−0.2466	—

GLCM, gray-level co-occurrence matrix; GLDS, gray-level difference statistics; GLRLM, gray-level run length matrix; GLZSM, gray-level zone size matrix; NGTDM, neighborhood gray-tone difference matrix; LHH = lowpass filter + highpass filter + highpass filter.

**Table 3:** Univariate and multivariate regression analysis for the Rad-score, clinical risk factors, and conventional PET features in the 70 LAPC patients.

Parameters	Univariate analysis		Multivariate analysis	
	HR (95% CI)	*p* value	HR (95% CI)	*p* value
Age	1 (1–1)	0.084		
Sex	0.99 (0.6–1.7)	0.98		
ECOG	0.89 (0.63–1.3)	0.5		
Tumor diameter	1.3 (1.1–1.5)	0.012	1.1 (0.86–1.4)	0.414
Location	1.1 (0.64–2)	0.68		
T stage	1.8 (1.3–2.4)	<0.01	0.99 (0.63–1.6)	0.97
N stage	1.7 (1.1–2.9)	0.031	1.2 (0.66–2.1)	0.61
CA19-9	1 (1–1)	0.063		
Chemotherapy	0.39 (0.22–0.69)	0.001	0.55 (0.26–1.2)	0.11
Dose	0.9 (0.85–0.95)	<0.01	0.47 (0.21–1)	0.056
SUVmax (early)	2.6 (0.88–7.5)	0.083		
SUVmean (early)	2.5 (0.85–7.1)	0.097		
MTV (early)	2.5 (0.65–9.3)	0.19		
TLG (early)	3.6 (1.1–1.2)	0.037	0.77 (0.095–6.3)	0.806
SUVmax (delay)	5.1 (1.6–17)	0.007	2.1 (0.1–43)	0.632
SUVmean(delay)	3.2 (1–9.9)	0.048	4 (0.075–210)	0.494
MTV (delay)	8.9 (2.1–3.7)	0.003	-	0.537
TLG (delay)	9.8 (2.5–38)	0.001	-	0.717
Rad_score (dual)	3.2 (2.1–5)	<0.001	4.1 (2.1–8.1)	<0.001

ECOG, eastern cooperative oncology group; CA19-9, carbohydrate antigen 19–9; dose, radiotherapy dose (Gy); MTV, metabolic tumor volume; TLG, total lesion glycolysis.

## Data Availability

Emails could be sent to the address wongfei@shu.edu.cn to obtain the shared data.

## Supplementary Materials

Supplementary Materials Supplementary Table 1: Statistics of the multidomain features. For the columns of “Modality,” the term “&” means the features are calculated for both domains. GLCM, gray-level co-occurrence matrix; GLDS, gray-level difference statistics; GLRLM, gray-level run length matrix; GLZSM, gray-level zone size matrix; NGTDM, neighborhood gray-tone difference matrix; LHH = lowpass filter + highpass filter + highpass filter. Supplementary Table 2: The results of Wilcoxon rank-sum test based on resampling.
