# Supplementary material for: Prognostic Evaluation Based on Dual-Time 18F-FDG PET/CT Radiomics Features in Patients with Locally Advanced Pancreatic Cancer Treated by Stereotactic Body Radiation Therapy
Source: J Oncol. 2022 Jul 14;2022:6528865. doi: 10.1155/2022/6528865 (PMC9303166; doi:10.1155/2022/6528865)
Supplement: Supplementary Materials — Supplementary Table 1: Statistics of the multidomain features. For the columns of “Modality,” the term “&” means the features are calculated for both domains. GLCM, gray-level co-occurrence matrix; GLDS, gray-level difference statistics; GLRLM, gray-level run length matrix; GLZSM, gray-level zone size matrix; NGTDM, neighborhood gray-tone difference matrix; LHH = lowpass filter + highpass filter + highpass filter. Supplementary Table 2: The results of Wilcoxon rank-sum test based on resampling. [file 6528865.f1.zip › Supplementary Table 1.docx]

| **Categories** | **Features** | | **Modality** |
| --- | --- | --- | --- |
| First-order statistics features | intensity histogram features | Standard variance, Entropy, Skewness, Kurtosis | PET&CT |
| Morphological features | Volume | | PET/CT |
|  | Solidity, Eccentricity, Longest diameter | |  |
| Texture features | 20 GLCM features (2nd-order): Energy, Auto correlation, Contrast, Correlation, Cluster prominence, Entropy, Cluster shade, Dissimilarity, Homogeneity, Maximum probability, Sum of squares, Sum average, Sum variance, Sum entropy, Difference variance, Difference entropy, Information measure of correlation1 (IMC1), Information measure of correlation2 (IMC2), Inverse difference normalized, Inverse difference moment normalized  13 GLRLM features (High-order): Short Run Emphasis, Long Run Emphasis, Gray Level Non-Uniformity, Run Length Non-Uniformity, Run Percentage, Low Gray Level Run Emphasis, High Gray Level Run Emphasis, Short Run Low Gray Level Emphasis, Short Run High Gray Level Emphasis, Long Run Low Gray Level Emphasis, Long Run High Gray Level Emphasis, Gray Level Variance, Run length Variance  4 GLDS features (1nd-order): Mean, Entropy, Contrast, Energy  5 NGTDM features (High-order): Coarseness, Contrast, Busyness, Complexity and Strength  13 GLZSM features (High-order): Small Zone Emphasis, Large Zone Emphasis, Gray Level Non-uniformity, Zone Size Non-uniformity, Zone Percentage, Low Gray Level Zone Emphasis, High Gray level Zone Emphasis, Small Zone Low Gray Level Emphasis, Small Zone High Gray-Level Emphasis, Large Zone Low Gray-Level Emphasis, Large Zone High Gray-Level Emphasis, Gray Level Variance and Zone Size Variance | | PET&CT |
| Wavelet | Texture features for four rotational-invariant groups of 1 level wavelet decomposition: LLL, LLH+LHL+HLL, LHH+HLH+HHL, and HHH | | PET&CT |
