# Supplementary material for: Prognostic Evaluation Based on Dual-Time 18F-FDG PET/CT Radiomics Features in Patients with Locally Advanced Pancreatic Cancer Treated by Stereotactic Body Radiation Therapy
Source: J Oncol. 2022 Jul 14;2022:6528865. doi: 10.1155/2022/6528865 (PMC9303166; doi:10.1155/2022/6528865)
Supplement: Supplementary Materials — Supplementary Table 1: Statistics of the multidomain features. For the columns of “Modality,” the term “&” means the features are calculated for both domains. GLCM, gray-level co-occurrence matrix; GLDS, gray-level difference statistics; GLRLM, gray-level run length matrix; GLZSM, gray-level zone size matrix; NGTDM, neighborhood gray-tone difference matrix; LHH = lowpass filter + highpass filter + highpass filter. Supplementary Table 2: The results of Wilcoxon rank-sum test based on resampling. [file 6528865.f1.zip › Supplementary Table 2.docx]

|  | **Early** | **Delay** | **Dual** |
| --- | --- | --- | --- |
| **Early** | 1 | - | - |
| **Delay** | p<0.01 | 1 | - |
| **Dual** | p<0.01 | p<0.01 | 1 |
